# An open label, multicenter clinical trial that investigated the efficacy and safety of leuprorelin treatment of central precocious puberty in Chinese children

**DOI:** 10.1097/MD.0000000000028158

**Published:** 2021-12-23

**Authors:** Xiaoping Luo, Ling Hou, Yan Zhong, Cheng You, Yu Yang, Xian Wu, Pin Li, Shasha Zhou, Wenjuan Qiu, Huiwen Zhang, Ying Liu, Ye Qian, Feihong Luo, Ruoqian Cheng, Yuhua Hu, Haihong Gong, Qing Wang, Zhuangjian Xu, Hongwei Du, Feiyu Lu, Junfen Fu, Xuefeng Chen, Winston Wang, Ziheng Guo

**Affiliations:** a Department of Pediatrics, Tongji Hospital, Tongji Medical College, Huazhong University of Science & Technology, Wuhan, China; b Department of Child Healthcare, Hunan Children's Hospital, Changsha, China; c Department of Endocrinology, Metabolism and Genetics, The Affiliated Children's Hospital of Nanchang University, Jiangxi Provincial Children's Hospital, Nanchang, China; d Department of Endocrinology, Shanghai Children's Hospital, Shanghai Jiao Tong University, Shanghai, China; e Shanghai Institute for Pediatric Research, Xinhua Hospital Affiliated to Shanghai Jiao Tong University, School of Medicine, Shanghai, China; f Department of Endocrinology, Children's Hospital of Capital Institute of Pediatrics, Beijing, China; g Department of Endocrinology, Metabolism and Genetics, Children's Hospital of Fudan University, Shanghai, China; h Department of Pediatrics, Jiangsu Province Hospital, Nanjing, China; i Department of Pediatrics, Affiliated Hospital of Jiangnan University, Wuxi, China; j Department of Pediatric Endocrinology, The First Bethune Hospital of Jilin University, Changchun, China; k Department of Endocrinology, The Children's Hospital of Zhejiang University School of Medicine, National Clinical Research Center for Child Health, Hangzhou, China; l Takeda Development Center Asia, Shanghai, China; m Takeda Medical Affairs, Takeda (China) International Trading Co., Ltd, Shanghai, China.

**Keywords:** bone age/chronological age ratio, central precocious puberty, gonadotropin-releasing hormone, leuprorelin, Tanner stage

## Abstract

**Background:**

Leuprorelin is an analog of gonadotropin-releasing hormone that is used for the therapy of central precocious puberty (CPP). The aims of this prospective, open label, multicenter clinical trial were to establish its efficacy and safety during long-term use.

**Methods:**

Patients, who were all children, were treated with 1.88 to 3.75 mg leuprorelin subcutaneously once every 4 weeks for a total of 96 weeks between 2015 and 2018. The primary endpoint was the rate of occurrence of adverse events (AEs) and the secondary endpoint was no progression in the Tanner stage or regression by week 96 compared to baseline.

**Results:**

A total of 307 CPP patients, 305 (99.3%) females and 2 males (0.7%), completed the 96-weeks of treatment. Due to limited data for male patients, they are not discussed in the efficacy results. Treatment-emergent AEs (TEAEs) were reported for 252 (82.1%) patients, mostly (79.5%) being mild or moderate and only 33 (10.7%) of patients experienced TEAEs related to leuprorelin therapy. The most frequent (>2%) drug-related TEAEs were injection site induration (4.6%, 14/307) and vaginal bleeding (2.3%, 7/305). After treatment, 83.5% of patients had regression or no progression in the Tanner stage (95% confidence interval: 78.68%, 87.62%) and the majority had decreased gonadotropin-releasing hormone-stimulated peak luteinizing hormone and follicle-stimulating hormone concentrations, as well as reduced sex hormone concentrations and a reduction in the bone age/chronological age ratio compared to baseline.

**Conclusions:**

The trial revealed that CPP was effectively treated in most patients who received leuprorelin for nearly 2 years. Any drug-related AEs were reported with low incidence (<5%) and were consistent with the known safety profile of leuprorelin.

**Trial registration:**

The trial was registered at ClinicalTrials.gov (registration number: NCT02427958).

## 1 Introduction

Precocious puberty refers to the presence of secondary sexual characteristics in boys <9 years of age and girls <8 years old.^[1,2]^ Central precocious puberty (CPP) is triggered by the early activation of the hypothalamic-pituitary-gonadal (HPG) axis, resulting in inappropriate release of gonadotropin-releasing hormone (GnRH) and the onset of puberty.^[3]^ The incidence of CPP has been estimated to be about 1:5000 to 1:10,000 people, with the female/male ratio being about 10:1.^[4]^ In China, the incidence of CPP has increased in recent years. Epidemiology studies have shown that the occurrence of CPP in children 6 to 9 years old in Zhejiang province is about 0.38%^[5]^ and 1% in Shanghai.^[6]^ The relative ratio of CPP in Shanghai of girls to boys is about 4–5 to 1.^[6]^

The GnRH analog (GnRHa) has been administered in clinics since its first synthesis in 1980 and has been found to downregulate the GnRH receptor, inhibit gonadotropin secretion and lower the gonadotropin concentrations to pre-adolescent levels,^[3,7–9]^ which prevents the further development of secondary sexual characteristics and finally inhibits the HPG axis. Correspondingly, rapid growth and bone maturation were inhibited.^[3]^ After years of clinical practice, GnRHa has become the standard treatment for CPP.^[1,3]^ Reported treatment-emergent adverse events (TEAEs) included allergic or local reactions to GnRHa preparations, sterile abscess formations, withdrawal bleeding due to falling estrogen concentrations, sporadic case reports of convulsions, as well as slipped capital femoral epiphysis in a small number of patients.^[2]^

Leuprorelin is a type of GnRHa widely used to treat CPP worldwide, including in China. However, the dose of GnRHa administered varies in different countries, ranging from 10 to 350 μg/kg of body mass.^[10,11]^ Leuprorelin was approved in China in 1998 for treating CPP with doses of 30 to 90 μg/kg of body mass, given subcutaneously every 4 weeks. However, the National Health Commission of the People's Republic of China guidelines for CPP treatment recommends doses >90 μg/kg of body mass.^[1,12]^ In 2013, the National Medical Products Administration in China approved a new leuprorelin dose of 30 to 180 μg/kg of body mass, given subcutaneously every 4 weeks.^[13]^ Approval was based on the results of trials in the USA and Europe and a retrospective study in Japan.^[14,15]^ To date, there is a lack of safety and efficacy data for leuprorelin use to treat CPP in China, in particular for long-term use.

Therefore, the primary aim of the trial was to collect safety data from CPP patients treated with leuprorelin at the newly approved dose in China. Efficacy data, including Tanner stage evaluation,^[16,17]^ height and body mass measurements, gonadotropin and sex hormone concentrations, bone mineral density (BMD) measurement, bone age (BA) assessments, and ultrasonography of the pelvis, were collected and evaluated.

## 2 Methods

**Figure 1:**
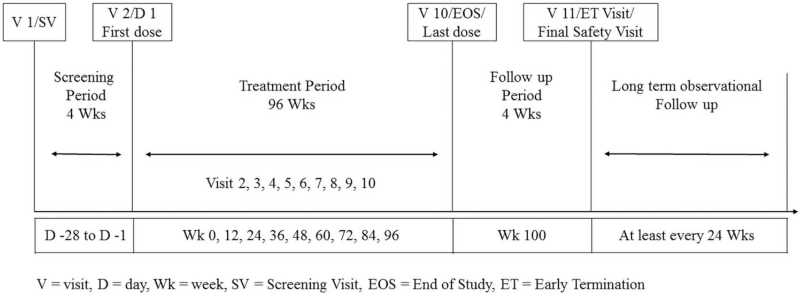
Dosage regimen protocol.

Three hundred seven children diagnosed with CPP in 11 medical centers in China were enrolled between 2015 and 2018 and treated with leuprorelin for 96 weeks. This study included a 4-week screening period, 96-week leuprorelin treatment, and 4-week safety follow-ups (Fig. 1). The trial was approved by ethics committees of Huazhong University of Science & Technology, Hunan Children's Hospital, Jiangxi Provincial Children's Hospital, Shanghai Children's Hospital, Xinhua Hospital Affiliated to Shanghai Jiao Tong University School of Medicine, Children's Hospital of Capital Institute of Pediatrics, Children's Hospital of Fudan University, Jiangsu Province Hospital, Affiliated Hospital of Jiangnan University, The First Bethune Hospital of Jilin University, and The Children's Hospital of Zhejiang University School of Medicine. All subjects and/or their legal representatives signed informed consent. The trial was registered at ClinicalTrials.gov (registration number: NCT02427958).

Eligible CPP patients whose body mass was ≥20 kg received a leuprorelin dose of 3.75 mg subcutaneously once every 4 weeks. Eligible CPP patients with body mass <20 kg received a leuprorelin dose of 1.88 mg subcutaneously at 4-week intervals. The dose was adjusted based on the condition of the patient and the investigator judgment. Stabilization of physical changes was evaluated every 12 weeks^[2]^ and included Tanner stage evaluation, height, and body mass measurements, BMD measurement, BA assessments, and ultrasonography of the pelvis. Other efficacy assessments included gonadotropin and sex hormone concentrations, while the safety assessment was mainly monitoring of adverse events (AEs).

### 2.1 Steroid hormone assays

Serum luteinizing hormone (LH), follicle-stimulating hormone (FSH), estradiol (E2), and testosterone and cortisol concentrations were measured by chemiluminescence (Access 2, Immunoassay System, Beckman Coulter Inc., USA; sensitivities 0.05 IU/mL, 0.2 IU/mL, 20 pg/mL, 0.35 nmol/L, 0.25 ng/mL, and 11 nmol/L, respectively). Serum adrenocorticotrophic hormone, dehydroepiandrosterone sulfate, and androstendione were determined by chemiluminescence assay (IMMULITE2000, Siemens Healthcare Diagnostic Inc., USA; sensitivity 5 pg/mL, 80 nmol/L, and 1.0 nmol/L, respectively). The serum concentration of 17-αOHP was determined using an enzyme-linked immunosorbent assay (DRG, EIA-1292, Germany; 0.034–20 ng/mL).

### 2.2 Endpoints

The primary endpoint was the rate of occurrence of TEAEs. The main secondary endpoint was the percentage of patients that experienced regression or no progression in the Tanner stage at week 96 compared with the Tanner stage at baseline. Additional endpoints were: the percentage of patients with suppression of peak LH and FSH to pre-pubertal concentrations in the stimulation test; the percentage of patients with suppression of basal E2 concentrations, in female patients or testosterone concentrations in male patients, to pre-pubertal concentrations at week 96; the percentage of patients with improvement in predicted adult height; percentage of patients with a decrease in the ratio of BA to chronological age (CA) at week 96 compared to baseline; and changes in body mass index (BMI) and BMD to week 96 from baseline.

The criteria for evaluating progress was an increase in the Tanner stage score for breast/genital or pubic hair, else it was assessed as being regressive or non-progressive. The upper limit value (ULV) for E2 was 20 pg/mL, for LH 2 U/L, and for FSH 6.7 U/L. The BA was evaluated using Tanner-Whitehouse 3^[18,19]^ standards and likely predicted duration of therapy of ≥2 years. The Bayley–Pinneau method was used to evaluate the predicted adult height.

### 2.3 Inclusion criteria

The main inclusion criteria were: secondary sexual characteristics appearance at age <8 years in girls or <9 years in boys, with persistent symptoms; CPP diagnosis confirmed; a basal concentration of LH >5.0 IU/L or a peak LH concentration >3.3 IU/L and LH/FSH >0.6 IU/L in the stimulation test; ultrasonographic evidence of gonadal development; advanced BA for ≥1 year with BA ≤11.5 years in females and ≤12.5 years in males; predicted adult females height <150 or <160 cm for males; and SDS <−2SD; or rapid growth of the BA/chronologic age >1.

### 2.4 Exclusion criteria

The main criteria for exclusion were: a patient had been given an experimental drug up to 30 days prior to potential enrollment; the patient had been treated with GnRHa in a previous trial; had abnormal laboratory values suggesting an underlying ailment or creatinine ≥1.5 mg/dL; alanine aminotransferase and/or aspartate aminotransferase >twice the normal higher limits; total bilirubin concentration >2.0 mg/dL, with aspartate aminotransferase/alanine aminotransferase elevated above normal levels; clinical signs or a previous history of kidney or thyroid disease; diagnosed with peripheral precocious puberty; a history of hypersensitivity or allergies to leuprorelin or related compounds including any excipients of the compound; a history or clinical manifestations of significant adrenal or thyroid diseases or intracranial tumor; or had a history of malignant disease.

### 2.5 Statistical methods

SAS software ver. 9.2 (SAS Institute, Inc., NC, USA) was used for all statistical analyses. Data are summarized for the following groups: males, females, and all patients. Continuous variables were analyzed including the mean, SD, median, minimum and maximum, and the number of observations. Categorical variables including the frequency and percentage of patients were also analyzed. Unless otherwise specified, all individual patient data are available in data listings. For binary data, the corresponding 95% confidence interval (CI) is given; 95% CIs were derived using the binomial “exact” Clopper–Pearson method. For all safety endpoints, the baseline was defined as the last non-deletion measure obtained before the first administration of the study drug. Safety data are summarized in the safety analysis set.

## 3 Results

### 3.1 Disposition of the patients

**Figure 2:**
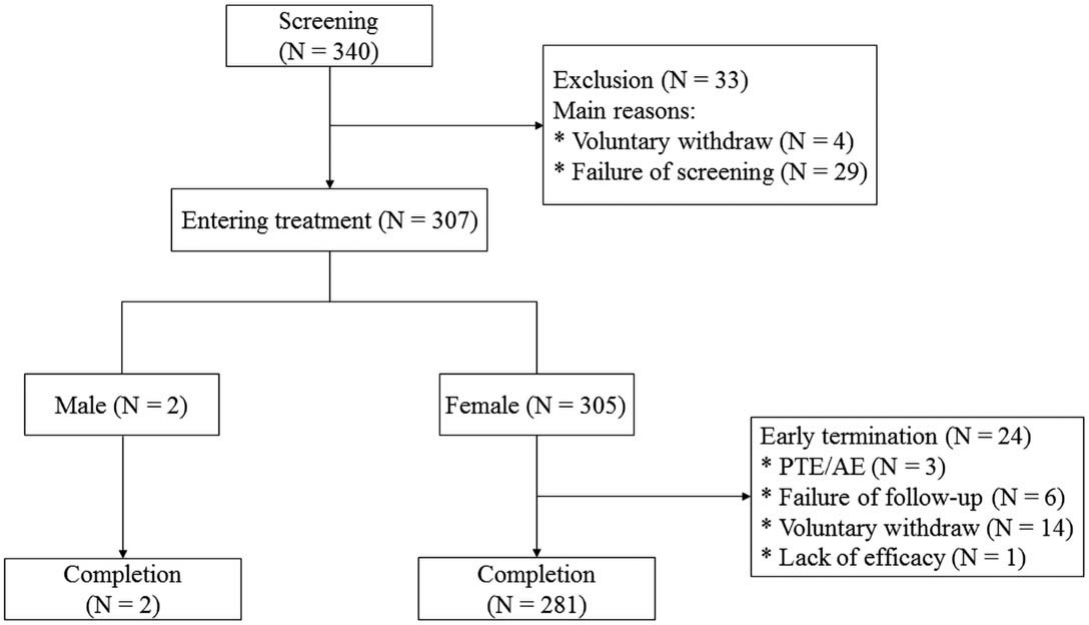
Disposition of patients.

A total of 340 patients were screened, of which 307 (90.3%) agreed to enter the treatment period from August 07, 2015 to November 23, 2018. A total of 283 patients completed the study and 24 (7.8%) terminated the trial prematurely. The main reasons for trial termination were voluntary withdrawal (N = 14), lost to follow-up (N = 6), AEs (N = 3), and poor efficacy (N = 1) (Fig. 2).

### 3.2 Demographics and baseline characteristics of patients

Female patients accounted for 99.3% (305/307) of the CPP population. Two male patients were enrolled in the trial; the sample size was too small for separate efficacy analyses, so these results are not discussed in the efficacy section of this paper. All efficacy results presented are based only on the 305 female patients, but safety results included the male patients. Detailed demographic and baseline characteristics of the enrolled patients are presented in Table 1.

**Table 1:** Demographics and other baseline characteristics (safety analysis set).

	Male (N = 2)	Female (N = 305)	All patients (N = 307)
Age (years)			
Mean (SD)	9.45 (0.354)	8.02 (0.930)	8.03 (0.935)
Height (cm)			
Mean (SD)	151.65 (5.869)	133.56 (6.971)	133.68 (7.108)
Body mass (kg)			
Mean (SD)	43.75 (3.182)	30.35 (5.249)	30.44 (5.346)
<20 kg, N (%)	0	5 (1.6)	5 (1.6)
≥20 kg, N (%)	2 (100)	300 (98.4)	302 (98.4)
Age when diagnosed as CPP (year)			
Mean (SD)	9.45 (0.354)	7.94 (0.977)	7.95 (0.982)
Tanner stage evaluation			
Genitals (male) and breast (female)			
I	1 (50.0)	2 (<1)	3 (<1)
II	1 (50.0)	171 (56.1)	172 (56.0)
III	0	120 (39.3)	120 (39.1)
IV	0	11 (3.6)	11 (3.6)
V	0	1 (<1)	1 (<1)
Pubic hair			
I	1 (50.0)	265 (86.9)	266 (86.6)
II	0	31 (10.2)	31 (10.1)
III	1 (50.0)	8 (2.6)	9 (2.9)
IV	0	0	0
V	0	1 (<1)	1 (<1)

Age = (the informed consent date − birth date + 1)/365.25. CPP = central precocious puberty, SD = standard deviation.

### 3.3 Exposure to the study drug

The maximum exposure time to the study drug was 687 days for all patients. Most of the patients (77.5%) were treated with leuprorelin for >672 days (96 weeks) and a total of 301 patients (98.0%) had >90% compliance (Table 2). A 96.1% of patients received an initial dose of leuprorelin ≥90 μg/kg of body mass. At week 96, most patients (62.6%) were still being given high doses.

**Table 2:** Summary of study drug exposure and compliance during the treatment period (safety analysis set).

	Female (N = 305)
Days of exposure	
Mean (SD)	652.3 (97.05)
Median	673.0
Min, max	1, 687
Exposure by category (days)	
1–84	3 (<1)
85–168	2 (<1)
169–252	2 (<1)
253–336	2 (<1)
337–420	4 (1.3)
421–504	1 (<1)
505–588	3 (<1)
589–672	51 (16.7)
>672	237 (77.7)
Study drug compliance (%)	
Mean (SD)	99.09 (2.418)
Median	100.00
Min, max	83.3, 100.0
<80%	0
80–90%	6 (2.0)
≥90%	299 (98.0)

SD = standard deviation.

### 3.4 Efficacy results

The incidence of regression or no progression in the Tanner stage at week 96 compared to baseline was 83.5% (238 of 285; 95% CI: 78.68%, 87.62%) and the incidence of progression in the Tanner stage at week 96 compared to baseline was 16.5% (47 of 285; 95% CI: 12.38%, 21.32%) for female patients (Table 3).

**Table 3:** Secondary endpoints.

Tanner staging	Female (N = 305)	
	N (%)	[95% CI]
Week 12		
N	302	
Regression/no progression	290 (96.0)	[93.16, 97.93]
Progression	12 (4.0)	[2.07, 6.84]
Week 96 (secondary endpoint)		
N	285	
Regression/no progression	238 (83.5)	[78.68, 87.62]
Progression	47 (16.5)	[12.38, 21.32]
Follow-up		
N	233	
Regression/no progression	233 (82.0)	[77.07, 86.33]
Progression	51 (18.0)	[13.67, 22.93]

Post stimulation test peak LH concentrations at week 96 were suppressed (peak value ≤ULV) in 90.4% of patients (253 of 280; 95% CI: 86.28%, 93.55%). Post stimulation test peak FSH concentrations at week 96 were also suppressed (peak value ≤ULV) in 95.4% of female patients (270 of 283; 95% CI: 92.27%, 97.53%). The percentage of female patients with suppression (E2 value ≤ULV) of basal E2 concentrations at week 96 was 59.4% (168 of 283; 95% CI: 53.39%, 65.14%).

The percentage of female patients with improvement in their predicted adult heights at week 96 compared to baseline was 64.6% (181/280; 95% CI: 58.73%, 70.24%). The mean change from baseline to week 96 of the predicted adult height was +2.19 (±5.263) cm. The percentage of patients exhibiting a decrease in the BA to CA ratio at week 96 compared to baseline was 94% (252 of 268; 95% CI: 90.49%, 96.55%). The mean change from baseline to week 96 in the BA to CA ratio was −0.11 ± 0.091). For BMI, the mean change from baseline to week 96 was 1.66 kg/m^2^, and the range was (−6.1, 9.4). For BMD, the mean change from baseline to the follow-up visits (∼week 100) was 0.041 g/cm^2^ (range −1.042, 2.788) (Table 4).

**Table 4:** Additional endpoints.

Parameter	Female n/N (%) [95% CI]
Simulated LH (U/L) peak value ≤ULV	253/280 (90.4) [86.28, 93.55]
FSH (U/L) peak value ≤ULV	270/283 (95.4) [92.27, 97.53]
Estradiol (pg/mL, female) ≤ULV	168/283 (59.4) [53.39, 65.14]
Testosterone (nmol/L, male) ≤ULV	-
Ratio of BA/CA	252/268 (94.0) [90.49, 96.55]
Predicted adult height	181/280 (64.6) [58.73, 70.24]
BMI	
Baseline (N)	305
Mean (SD)	16.93 (2.0)
Week 96 (N)	285
Mean (SD)	18.43 (2.4)
Change from baseline, mean (SD)	1.66 (1.6)
BMD	
Baseline (N)	181
Mean (SD)	0.48 (0.2)
Follow up until 100th week	170
Mean (SD)	0.52 (0.4)
Changes from baseline, mean (SD)	0.04 (0.4)

BA = bone age, BMD = bone mineral density, BMI = body mass index, CA = chronological age, FSH = follicle-stimulating hormone, LH = luteinizing hormone, SD = standard deviation, ULV = upper limit value.

### 3.5 Safety results

A total of 252 (82.1%) patients experienced at least one TEAE. The majority of patients (71.3%) experienced TEAEs that were not related to the study drug. Most of the TEAEs (79.5%) were mild (69.4%) or moderate (10.1%) in nature.

The most frequently reported TEAEs that occurred in ≥5% of patients were infections of the upper respiratory tract, cough, pyrexia, nasopharyngitis, bronchitis, pharyngitis, and tonsillitis. Of the TEAEs that were assessed as being drug-related, the most common were injection site induration (4.6%) and vaginal bleeding (2.3%). The incidence of other events was <1% (Table 5). In the present trial, 12 patients experienced 21 cases of serious AEs, but none were related to the study drug. One of the patients withdrew early from the trial because of a tibial fibula fracture and no patient died during the trial period. No observable trends were detected in the clinical laboratory, vital signs, or electrocardiogram data.

**Table 5:** Primary endpoint: TEAE incidence rate and drug-related TEAEs (safety analysis set).

	Male (N = 2, PY = 3.838)		Female (N = 305, PY = 568.307)		Total (N = 307, PY = 572.145)	
	Events	Patients (%)	Events	Patients (%)	Events	Patients (%)
Treatment emergent AEs	7	1 (50.0)	1139	251 (82.3)	1146	252 (82.1)
At least one related	2	1 (50.0)	62	32 (10.5)	64	33 (10.7)
Mild	7	1 (50.0)	1056	212 (69.5)	1063	213 (69.4)
Moderate	0	0	68	31 (10.2)	68	31 (10.1)
Severe	0	0	15	8 (2.6)	15	8 (2.6)
Leading to discontinuation of the study drug	0	0	7	2 (<1)	7	2 (<1)
SAE during TEAE	0	0	21	12 (3.9)	21	12 (3.9)
Drug-related TEAEs during the treatment phase						
Injection site induration	2 (52.10)	1 (50.0)	39 (6.86)	13 (4.3)	41 (7.17)	14 (4.6)
Vaginal hemorrhage	0	0	8 (1.41)	7 (2.3)	8 (1.40)	7 (2.3)
Injection site reaction	0	0	3 (0.53)	3 (<1)	3 (0.52)	3 (<1)
Subcutaneous abscess	0	0	3 (0.53)	2 (<1)	3 (0.52)	2 (<1)
Obesity	0	0	2 (0.35)	2 (<1)	2 (0.35)	2 (<1)
Others	0	0	7 (1.26)	7 (<1)	7 (1.19)	7 (<1)

Percentages are based on the number of patients in the safety data set for each gender. For the incidence rate per 100 person-years, percentages are based on the number of AEs. N = number of patients in the analysis population, PY = total time at risk in years.

## 4 Discussion

This was a large, open prospective clinical trial conducted in China to determine the efficacy and safety of leuprorelin for the long-term therapy of CPP. In the trial, 305 female children diagnosed with CPP received leuprorelin once a month for 96 weeks. Most patients began and maintained their therapy at a fixed dose of 3.75 mg, which produced serum concentrations ≥90 μg/kg of body mass.

For more than 30 years, GnRHa administration has been the gold standard used to treat CPP. This treatment aims to improve the height of the eventual adult by inhibiting the HPG axis, preventing the progression of puberty, and delaying bone maturation.^[2]^ Two-phase 3 clinical trials were carried out in the USA to establish the efficacy and safety of leuprorelin for CPP therapy. The first trial was an open, non-comparative study using both daily subcutaneous and reservoir dosage forms in accordance with the USA investigational new drug and protocol of each investigator's study.^[14]^ The second was a phase 3, open, non-comparative trial, but was conducted in accordance with the sponsor's protocol and the USA investigational new drug.^[14]^ The 2 trials were carried out in 39 research centers. A total of 226 children were evaluated for efficacy and 365 assessed for safety. Children with CPP were treated with leuprorelin subcutaneous injections or a reservoir for up to 4.9 and 2.7 years, respectively. The maximum duration of treatment was 5.3 years (1 child received 2 dosage forms of the medication). In these trials, the recommended initial dose was 50 or 300 μg/kg/day once a month. In both studies, leuprorelin was shown to be safe and effective in reducing gonadotropin and sex hormone serum concentrations in children with CPP. In phase 3 clinical trial conducted in 25 research centers in Germany, the 3.75 mg treatment regimen was shown to have the best efficacy and safety and is considered to be the European-approved CPP treatment regimen.

Our results showed that most of the patients had decreased LH and FSH peak concentrations in the GnRH stimulation test, as well as decreased sex hormone concentrations, and exhibited regression or non-progression of the Tanner stage, thus achieving inhibition of CPP. The predicted adult height increased and the BA/CA ratio decreased. Therefore, treatment with a dose of 3.75 mg leuprorelin every 4 weeks was consistent with the previously reported conventional therapeutic effect.^[5,20–22]^

In the present trial, the BMIs of the children increased after 96 weeks of leuprorelin therapy compared to baseline. While a number of studies have shown that BMI can increase after treatment, a number of others reported that the increase in the BMI standard deviation score during treatment appeared to be a transient phenomenon. After cessation of therapy, no significant correlations between being overweight, obese, treatment, or CPP duration before GnRHa treatment were found.^[23]^

Treatment with GnRHa slows mineral accumulation, but the BMD did not differ significantly from that of late adolescent peers after withdrawal, with the BMD in all patients being within the normal range.^[24,25]^ The present trial results revealed that after 96 weeks of GnRHa treatment, that BMD values in children with CPP were largely unchanged from baseline. Decades of clinical experience have shown that treatment with GnRHa is safe and effective,^[2]^ with few adverse reactions, but the association between most reported AEs and GnRHa administration nevertheless remained unclear.

The majority of patients in this large prospective trial received medication for 96 weeks, of which 71.3% exhibited TEAEs that were considered to be unrelated to the study drug, and most of the reported AEs were mild. Of those that were related to the study drug, the most common were injection site induration and vaginal bleeding. The reports of induration at the injection site subsided spontaneously with no special treatment being required. About 10% to 15% of patients have been reported in the literature to have injection site reactions (occasionally presenting as sterile abscesses), but generally the drug was well tolerated.^[26,27]^

The incidence of vaginal bleeding all occurred once during the first 1 to 2 months of treatment. It is generally believed that girls with a pronounced endometrium lining may experience vaginal bleeding after the start of GnRHa treatment due to a drop in estrogen concentration. If bleeding persisted after 2 months of treatment, it suggests that gonadotropin inhibition had not occurred or there was another mechanism involved.^[2]^

The limitations of the present trial were the small number of only 2 male patients involved and that the enrolled CPP children were not further divided into slow progression and rapid progression types. In addition, the participating 11 children's medical centers were located in relatively developed areas, which may not accurately reflect the general treatment status of early puberty in China. However, despite these limitations, this paper provides valuable data to guide the future treatment of Chinese children with CPP.

## 5 Conclusions

In this 96-week trial of CPP patients, leuprorelin was well tolerated. Any drug-related AEs occurred at a low incidence (<5%). Safety observed in the trial was consistent with the known safety profile of leuprorelin and there were no concerns about long-term administration. Leuprorelin was shown to have a good efficacy and safety profile for CPP therapy in China when administered at a dose of 3.75 mg once every 4 weeks.

## Acknowledgments

We thank the Clinical Science and Operation Team of the Takeda Development Center Asia for their efforts in the research process and Takeda China Medical Affairs for their contribution to the publication. We also appreciate the contribution of Eric Lloyd and the Takeda Research and Development Center America for their valuable support of the statistical analyses.

## Author contributions

Authors in each center were involved in the design of the study and the collection of data. All authors were involved in drafting and critically revising the manuscript. All authors take responsibility for the integrity of the data and its accuracy.

**Conceptualization:** Xiaoping Luo, Ling Hou, Yan Zhong, Cheng You, Yu Yang, Xian Wu, Pin Li, Shasha Zhou, Wenjuan Qiu, Huiwen Zhang, Ying Liu, Ye Qian, Feihong Luo, Ruoqian Cheng, Yuhua Hu, Haihong Gong, Qing Wang, Zhuangjian Xu, Hongwei Du, Feiyu Lu, Junfen Fu, Xuefeng Chen, Winston Wang, Ziheng Guo.

**Data curation:** Xiaoping Luo, Ling Hou, Yan Zhong, Cheng You, Yu Yang, Xian Wu, Pin Li, Shasha Zhou, Wenjuan Qiu, Huiwen Zhang, Ying Liu, Ye Qian, Feihong Luo, Ruoqian Cheng, Yuhua Hu, Haihong Gong, Qing Wang, Zhuangjian Xu, Hongwei Du, Feiyu Lu, Junfen Fu, Xuefeng Chen, Winston Wang, Ziheng Guo.

**Project administration:** Xiaoping Luo.

**Supervision:** Xiaoping Luo, Cheng You, Xian Wu.

**Validation:** Xiaoping Luo, Ling Hou, Yan Zhong, Cheng You, Yu Yang, Xian Wu, Pin Li, Shasha Zhou, Wenjuan Qiu, Huiwen Zhang, Ying Liu, Ye Qian, Feihong Luo, Ruoqian Cheng, Yuhua Hu, Haihong Gong, Qing Wang, Zhuangjian Xu, Hongwei Du, Feiyu Lu, Junfen Fu, Xuefeng Chen, Ziheng Guo.

**Visualization:** Xiaoping Luo, Ling Hou, Yan Zhong, Yu Yang, Pin Li, Shasha Zhou, Wenjuan Qiu, Huiwen Zhang, Ying Liu, Ye Qian, Feihong Luo, Ruoqian Cheng, Yuhua Hu, Haihong Gong, Qing Wang, Zhuangjian Xu, Hongwei Du, Feiyu Lu, Junfen Fu, Xuefeng Chen, Winston Wang, Ziheng Guo.

**Writing – original draft:** Xiaoping Luo, Ling Hou.

**Writing – review & editing:** Xiaoping Luo, Ling Hou, Yan Zhong, Cheng You, Yu Yang, Xian Wu, Pin Li, Shasha Zhou, Wenjuan Qiu, Huiwen Zhang, Ying Liu, Ye Qian, Feihong Luo, Ruoqian Cheng, Yuhua Hu, Haihong Gong, Qing Wang, Zhuangjian Xu, Hongwei Du, Feiyu Lu, Junfen Fu, Xuefeng Chen, Winston Wang, Ziheng Guo.
